# Cardiotoxicity Associated with Venlafaxine—Defining Features in a Series of Five Cases and a Call for Proactive Monitoring

**DOI:** 10.3390/jcm14082792

**Published:** 2025-04-18

**Authors:** Bujana Batusha Sopi, Keiko Yonekawa, Stefan Russmann, Jasminka Bernheim, Stefano Caselli, Christian Schmied, Helene Hammer, Anna Lam, Christine Attenhofer Jost

**Affiliations:** 1 HerzGefässZentrum Im Park, Hirslanden Klinik Im Park, 8038 Zürich, Switzerland; bujana.batusha@gmail.com (B.B.S.); jasminka.bernheim@hirslanden.ch (J.B.); stefano.caselli@hirslanden.ch (S.C.); helene.hammer@hirslanden.ch (H.H.); anna.lam@spitalstsag.ch (A.L.); 2 Drugsafety.ch, 8703 Küsnacht, Switzerland; stefan.russmann@hirslanden.ch; 3 University of Nicosia Medical School, Engomi 2408, Cyprus

**Keywords:** venlafaxine, cardiomyopathy, cardiotoxicity, heart failure, arrhythmias

## Abstract

**Background/Objectives:** Venlafaxine (VEN) is a serotonin and norepinephrine reuptake inhibitor (SNRI) antidepressant. Arterial hypertension (HTN), heart failure (HF), and arrhythmias are side effects of VEN. Cardiotoxicity (CTOX) as a feature of VEN-associated side-effects has only rarely been described. **Methods:** We conducted a search of our database for cases of VEN-associated CTOX, analyzing symptoms, echocardiographic findings, and laboratory results. **Results:** We identified five patients (three females, two males) with VEN-associated CTOX, aged 51 to 87 years at presentation. VEN dose was 150 and 375 mg daily and treatment duration was 1.5 to 15 years. Presenting features were HTN in three, “hypertrophic cardiomyopathy” in two, heart failure in three, and atrial fibrillation in three patients. Symptoms and signs of CTOX were reversible in all patients after discontinuation or dose reduction of VEN, suggesting a causal relationship between VEN and CTOX. **Conclusions:** VEN-associated CTOX can occur and progress to severe cardiomyopathy or heart failure. Potential risk factors include cardiac sympathetic stimulation, high VEN dosage, and prolonged treatment duration; however, CTOX may also occur at standard doses. Therefore, patients taking VEN should be routinely monitored for signs of cardiotoxicity, including monitoring of serum concentrations of VEN.

## 1. Introduction

Venlafaxine (VEN) is a serotonin–norepinephrine reuptake inhibitor (SNRI) that was introduced into clinical practice in 1993. It is indicated for various psychiatric disorders including depression, anxiety and panic disorders, and social phobias [1,2,3]. The antidepressant effect of VEN is believed to be primarily attributed to increased cerebral concentrations of the neurotransmitters serotonin and norepinephrine. Furthermore, VEN is also a weak inhibitor of dopamine reuptake.

Currently, VEN is commonly used as first-line treatment, or a second-line option for patients who do not respond to selective serotonin reuptake inhibitors (SSRIs). The usual maintenance dose ranges from 75 to 225 mg/day, but doses up to 375 mg daily have been approved, particularly for patients transitioning from SSRIs to SNRIs [4]. VEN exhibits dose-dependent pharmacodynamics: at lower doses, it primarily inhibits serotonin reuptake, while at higher doses (≥150 mg/day), it additionally inhibits norepinephrine reuptake and exerts weak inhibitory effects on dopamine reuptake. This dose-dependent effect allows for individualized therapy based on the severity of depression and the patient’s response to treatment.

Common non-cardiac side effects include nausea, dizziness, dry mouth, sweating, insomnia, drowsiness, decreased appetite, sexual dysfunction, headaches, and withdrawal symptoms upon discontinuation.

There are concerns regarding the cardiovascular safety profile of VEN. Documented cardiovascular complications include tachycardia, arterial hypertension, left ventricular hypertrophy, arrhythmia, and prolongation of the QT interval. These side effects are of particular importance as they may heighten the risk of serious cardiovascular events such as heart failure and sudden cardiac death.

Given these potential risks, the safety of VEN, especially with long-term use and at high doses, is under surveillance. Cardiac toxicity has been a major concern since 2004, leading to restrictions on SNRI use in the UK from 2004 until 2006 [5].

The aim of this publication is to shed light on the cardiotoxic effects of VEN. We present an evaluation of five patients treated in our clinic for cardiovascular side effects potentially related to VEN use. These case studies aim to raise awareness of VEN-associated cardiac toxicity (CTOX), identify potential risk factors, and develop strategies for monitoring and treating these side effects.

## 2. Methods

We conducted a retrospective analysis of our echocardiographic database for cases of VEN-associated CTOX, identifying five patients who displayed symptoms of VEN toxicity that were reversible upon reducing or discontinuing VEN. The database used for this study is internally managed and not publicly available due to confidentiality and data protection regulations. Data collection additionally included clinical records, laboratory results, echocardiographic findings, ECG reports, and patient-reported health questionnaires. These data sources were thoroughly reviewed and validated by the treating physician and study nurse prior to being entered into a password-protected, encrypted Excel database.

Side effects were documented by both treating physicians and study nurses, drawing on clinical observations, patient self-reports, and laboratory findings. All side effects were initially recorded in standardized case report forms (CRFs) and subsequently transferred into the database using appropriate patient coding to ensure confidentiality and anonymity.

We reviewed all available clinical data, including duration and dosage of VEN and changes in symptoms, ECG, transthoracic echocardiographic findings, changes in NT-proBNP levels where available, and correlation with kidney function. All patients had at least 2 echocardiographic examinations. The echocardiographic examinations were complete and included assessment of left ventricular size, left ventricular hypertrophy, left ventricular global longitudinal strain (GLS), diastolic function, atrial size, and estimation pulmonary artery pressure, as well as assessment of valvular function according to published guidelines.

Inclusion criteria comprised patients undergoing cardiological evaluation, including an echocardiographic examination at our center (HerzGefässZentrum, Hirslanden Klinik Im Park), who were treated with venlafaxine and demonstrated potential signs of cardiotoxicity—such as heart failure, tachyarrhythmias, QT interval prolongation, arterial hypertension, or echocardiographic abnormalities—that could not be attributed to other causes and were temporally associated with venlafaxine therapy, and that were reversible. Exclusion criteria were defined as the inability or unwillingness of patients to provide informed consent.

## 3. Case Descriptions

### 3.1. Patient #1

A 52-year-old woman was prescribed VEN 150 mg/day for chronic treatment-resistant migraine. After 1.5 years, she presented to our clinic with dyspnea and arterial hypertension (170/80 mmHg). Other medications included estrogen gel, progesterone capsule, and fremanezumab. Known medical issues included migraines/headaches and pregnancy hypertension.

Cardiovascular risk factors included arterial hypertension and dyslipidemia. Family history was negative for heart muscle disease or hypertrophic cardiomyopathy.

**Figure 1:**
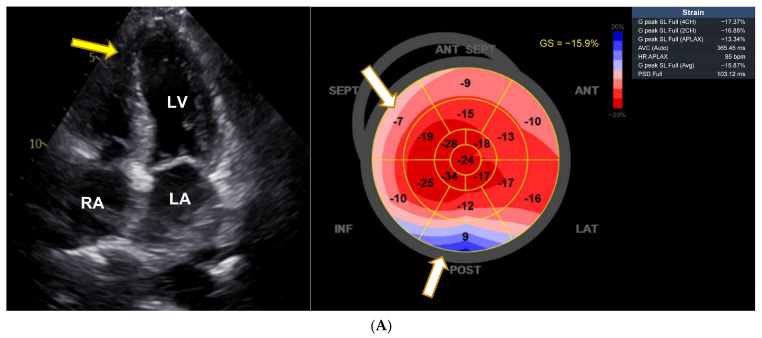

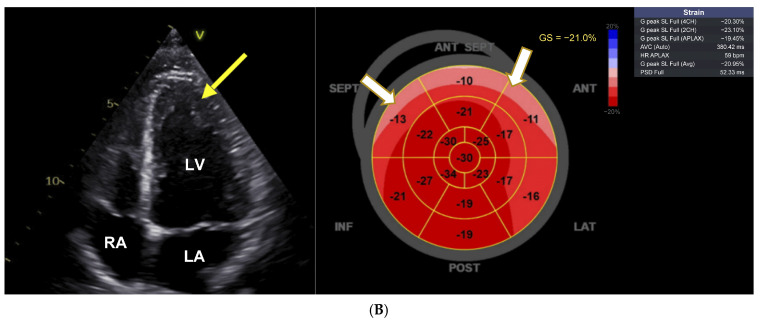
Pronounced cardiac anomaly showing features of hypertrophic cardiomyopathy. (**A**) Echocardiographic changes with apical hypertrophy and apical sparing pattern with decrease in strain in basal segments (white arrows). LA = left atrium, LV = left ventricle, RA = right atrium; white arrow points to left ventricular hypertrophy. Average GLS as shown on the right side is diminished and shows apical sparing (white arrows). (**B**) Reversal of left ventricular hypertrophy and apical sparing pattern after dose reduction of venlafaxine. LA = left atrium, LV = left ventricle, RA = right atrium; yellow arrow points to left ventricular hypertrophy. Average GLS as shown on the right side has now almost completely normalized (−21.9%), with some remaining decrease in basal septal, anteroseptal, and anterior segments (white arrows).

There was normal systolic and diastolic function, normal renin, aldosterone, adrenalin, and noradrenaline levels measured in serum, methoxytyramine was massively elevated (309 nmol/mL, normal range 0.59–4.19), and MRI showed no kidney or adrenal abnormalities. Echocardiography revealed signs of apical hypertrophic cardiomyopathy and decrease in global longitudinal strain (GLS) to −15.9% (Figure 1A). VEN serum concentration was not determined. VEN-associated CTOX was suspected and her migraine treatment switched from VEN to fremanezumab. Subsequently, within one year, the patient showed almost normal blood pressure values, normalization of ECG changes, and an increase in GLS to −21%. The echocardiographic signs of left ventricular hypertrophy had regressed (Figure 1B), and blood pressure had decreased to 143/84 mmHg. She had no dyspnea anymore, and her methoxytyramine levels had completely normalized.

### 3.2. Patient #2

A 51-year-old woman with chronic anorexia nervosa (43 kg, 167 cm, BMI 15.4 kg/m^2^) had been treated with 150 mg/day VEN for her anorexia for 15 years. She developed arterial hypertension (151/100 mmHg) and was sent for further cardiac evaluation.

Other medications included valsartan and amlodipine. Besides anorexia and hypertension, no other medical issues were reported. The only cardiovascular risk factors were hypertension and a positive family history of carotid artery stenosis in the mother, while there was a negative family history of heart disease. The patient only had a history of Raynaud’s syndrome fingers.

**Figure 2:**
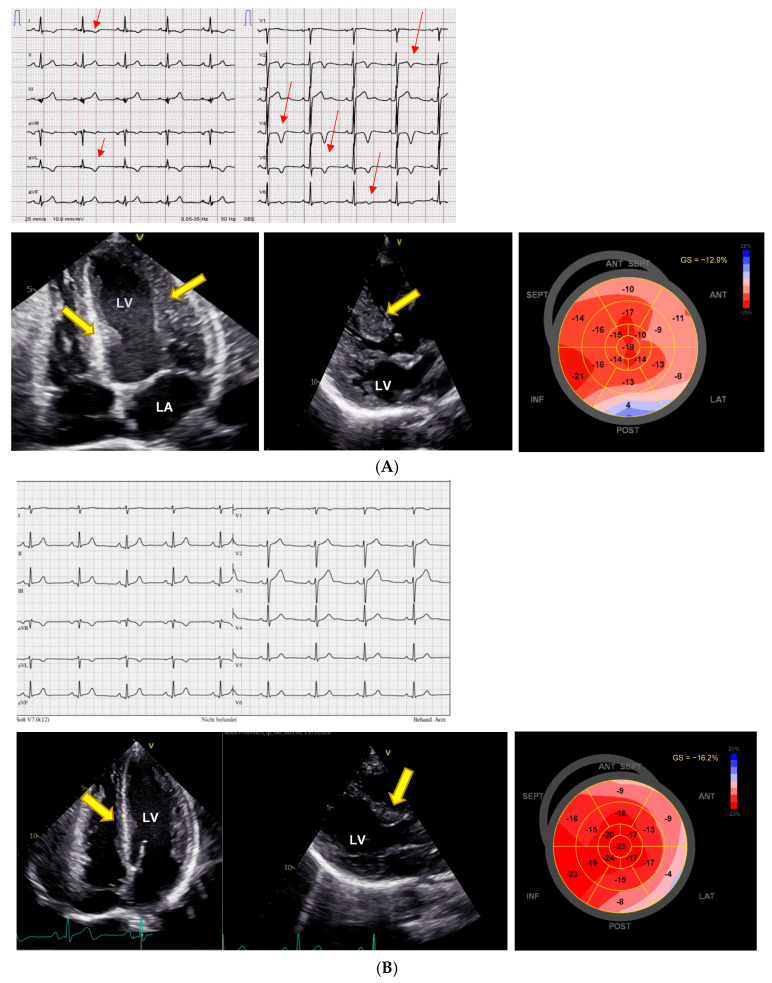
Reversible hypertrophic cardiomyopathy after dose reduction. (**A**) ECG and echocardiography with pronounced changes resembling hypertrophic cardiomyopathy. First cardiac evaluation, May 2023. The ECG shows normal sinus rhythm with a QTc interval of 455ms and negative T-waves resembling HCM (red arrows). Transthoracic echocardiography during therapy with venlafaxine 150 mg shows severe left ventricular hypertrophy (yellow arrows) with a preserved left ventricular ejection fraction of 60%, and a severely diminished left ventricular global longitudinal strain of −12.9%. LV = left ventricle, LA = left atrium. (**B**) Reversal of ECG changes and regression left ventricular hypertrophy (yellow arrows) after 1.5 years as well as improvement of left ventricular global longitudinal strain to −16.2%.

NT-proBNP was 226 ng/L (normal < 121 ng/L), there were no signs of pheochromocytoma, and renal function was normal. She had no cardiac symptoms, but there were ECG changes (Figure 2A), and transthoracic echocardiography revealed pronounced LVH and severe reduction in the LV GLS to −13%. A pharmacogenetic investigation found a CYP2D6 intermediate metabolizer phenotype (AS 1.0, genotype *1/*4.001) IM, and CYP2C19 ultrarapid metabolizer phenotype (genotype *17/*17). VEN dose was reduced to 37.5 mg/day as the patient refused to stop it completely, and under the reduced dose serum concentrations were low-normal (VEN 0.12 µmol/L; O-desmethyl-VEN 0.31 µmol/L, and VEN + O-desmethyl-VEN 0.43 µmol/L (normal 0.36–1.44 µmol/L)). Subsequently, the ECG normalized, and echocardiographic abnormalities regressed (Figure 2B). Cardiac evaluation in November 2024 under Venlafaxine 75 mg shows regression of LV-hypertrophy (yellow arrows), a LVEF of 54%, and an improvement in the LV strain (−16.2%).

### 3.3. Patient #3

A 77-year-old woman was regularly seen at our cardiology clinic for prior mitral valve replacement (mechanical valve). The family history was negative for heart disease, and no other cardiovascular risk factors were present. Coronary artery disease had been ruled out through normal coronary angiography. She had a history of prior ergotamine use that led to mitral valve disease, as well as a previous transient ischemic attack. Other medications included amiodarone, spironolactone, candesartan, atorvastatin, torasemide, apixaban, and lorazepam. During follow-up after mitral valve replacement, she had a normal left ventricular ejection fraction (61%) and no relevant arrhythmias.

She was started on VEN for severe depression and eventually received an unusually high dose of 375 mg/day, and VEN and O-desmethyl-VEN levels were far above the upper normal range (6.83 µmol/L, normal 0.36–1.44 µmol/L). The patient was initially treated with VEN 150 mg/day from October 2019, with an escalation to 300 mg/day in April 2020. In October 2020, she developed heart failure, presenting with a marked reduction in LVEF to 27%, an elevated NT-proBNP of 3492 ng/L, and paroxysmal atrial fibrillation. Despite recommendations to discontinue VEN, the patient opted for a dose reduction to 150 mg/day. Over the following year, a significant improvement was observed, with LVEF recovering to 66%, NT-proBNP normalizing, and paroxysmal atrial fibrillation occurring less frequently. Subsequently, VEN was titrated up to 375 mg/day, and by 11 December 2023, the patient experienced recurrent heart failure, evidenced by a decline in LVEF to 42% and NT-proBNP elevation to 3464 ng/L, though, notably, paroxysmal atrial fibrillation was absent. Following a dose reduction to 150 mg/day by 27 December 2023, another remarkable recovery was noted, with LVEF improving to 62%, NT-proBNP decreasing to 849 ng/L, and paroxysmal atrial fibrillation becoming less frequent.

The course of patient #3 is also summarized in Table 1.

### 3.4. Patient #4

**Figure 3:**
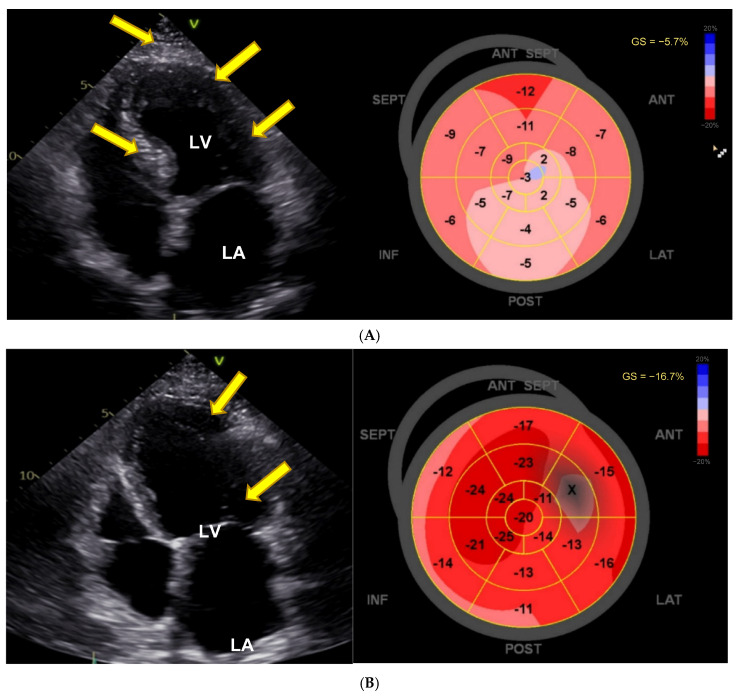
Severe impairment of LV ejection fraction parallel to reduction in LV strain, reversible after dose reduction. (**A**) During high-dose venlafaxine 187.5 mg therapy there is left ventricular (LV) hypertrophy (yellow arrows), a significantly reduced left ventricular ejection fraction (LVEF) of 20%, and severely impaired left ventricular global longitudinal strain (GLS) of −5.7%, indicating marked systolic dysfunction. LV = left ventricle, LA = left atrium. (**B**) Under venlafaxine 75 mg, a regression of left ventricular (LV) hypertrophy (yellow arrow) was observed, with an improvement in LV ejection fraction (LVEF) to 49% and an increase in left ventricular global longitudinal strain (GLS) to −16.7%.

An 82-year-old man had been taking VEN for more than 8 years in a dose of up to 187.5 mg/day. LVEF had been documented as normal in the past. In spring 2023, he presented with severe heart failure, pleural effusions, edema, renal failure, massive increase in NT-proBNP (22,743 ng/L, age-adjusted normal < 450 ng/L), a dilated LV with severely diminished LVEF of 20% and impaired LV GLS (Figure 3A). Coronary angiography ruled out significant coronary artery disease. Other medications included bisoprolol, sacubitril/valsartan, torasemide, empagliflozin, tamsulosin, insulin, immunoglobulin treatment (i.v., every 2 weeks), and warfarin. The patient was also diabetic and had a history of necrotizing myositis, possibly due to statin treatment (thus the immune treatment), along with a history of sensory polyneuropathy, diabetes, and chronic issues related to previous subarachnoid hemorrhage. VEN was subsequently reduced to 75 mg/day. At the last follow-up in November 2024, he was asymptomatic; the biplane LVEF was 56% (normal 52–72%), and LV strain was borderline with a GLS of −16.7% (Figure 3B). NT-proBNP was normal at 227 ng/L. VEN serum concentrations were within normal limits (VEN 0.36 µmol/L, O-Desmethyl-VEN 0.18 µmol/L, VEN + O-Desmethyl-VEN 0.53 µmol/L; normal range 0.36–1.44 µmol/L). There was also stage 3b renal impairment, with an estimated glomerular filtration rate (eGFR) of 37 mL/min/m^2^.

### 3.5. Patient #5

An 87-year-old man with a history of coronary artery disease, previous revascularization, and a pacemaker implantation for second-degree AV block (Mobitz) had been taking VEN 150 mg/day for 4.3 years. Other medications included rivaroxaban, candesartan, rosuvastatin, mirtazapine, dutasteride, and empagliflozin. Cardiovascular risk factors included dyslipidemia, arterial hypertension, and a questionable history of heart disease; his mother had died at the age of 38 years, possibly due to congenital heart disease, while the family history was otherwise negative for heart disease. He developed worsening LV heart failure with a decrease in LVEF to 37% and significant LV hypertrophy. Venlafaxine was reduced to 75 mg/day. Subsequently, LV strain and NT-proBNP improved (Table 2), and signs of cardiac decompensation resolved.

A summary for these five patients with symptoms and signs of VEN-associated CTOX is presented in Table 3 and Table 4.

## 4. Discussion

We identified five patients with pronounced symptoms and signs of severe VEN-associated CTOX. The spectrum of cardiac changes involves ECG changes, hypertension, echocardiographic images resembling hypertrophic cardiomyopathy, atrial arrhythmias, and/or severe heart failure. After VEN was stopped or decreased, cardiac changes were almost completely reversible in all five patients (“positive dechallenge”), which is an important formal criterium for a probable causal relationship in standardized causality assessment of suspected adverse drug reactions.

In line with its mechanism of action, many known adverse effects of VEN appear to be catecholamine-related. Therefore, it is not surprising that several cardiac adverse effects have been described in association with VEN, ranging from frequent hypertension (36%) to rare occurrences of heart failure and Takotsubo syndrome [6,7,8]. VEN may also cause a pronounced increase in catecholamines or metanephrine, which can mimic pheochromocytoma. For example, there is a report of a 71-year-old woman taking 150 mg/d of VEN daily, in which the normetanephrine levels in the 24 h urine were clearly elevated and normalized after withholding VEN [9]. In line with this report, one of our patients (#1) had extremely elevated metanephrine levels, which normalized after discontinuing VEN. Considering the mechanism of action of SNRIs and the role of catecholamines in cardiotoxicity, one can assume at least a partial dose-dependency of VEN-associated CTOX. It is therefore reasonable to suggest that a dose reduction may be sufficient to minimize the cardiac adverse effects of VEN. Indeed, this assumption seems to be confirmed by the fact that, in four of the patients presented, we observed major improvements or even complete remission after at least a 50% dose reduction of VEN.

In two of our patients, electrocardiographic and echocardiographic changes resembled hypertrophic cardiomyopathy. To our knowledge, this has only been described in one patient with spinocerebellar ataxia taking VEN 150 mg/day, who experienced heart failure [10]. However, these signs of heart failure disappeared, and hypertrophy regressed despite the continuation of VEN therapy [10]. In any patient taking VEN, signs of cardiotoxicity should be considered potentially related to VEN treatment. Heart failure due to the intake of VEN has been documented in the literature in several reports. Singh reported a 37-year-old alcoholic with acute heart failure, an NT-proBNP level of 4921 ng/L, moderate renal insufficiency, sinus tachycardia at 117 bpm, elevated blood pressure, and a left ventricular ejection fraction of 25% without signs of coronary artery disease [11]. This patient had been taking VEN 300 mg/day for four years. Heart failure occurred in three of our five patients, and in all cases, it was reversible after a reduction in the VEN dose. In a similar report of a life-threatening episode of heart failure was observed in a 53-year-old female following a VEN and oxazepam overdose. There was QRS broadening, tachycardia at 123 beats per minute, a severe decrease in left ventricular ejection fraction to less than 20%, and elevations of NT-proBNP and troponin in the absence of coronary heart disease [12]. The patient recovered after ECMO and hemodialysis.

Prolongation of QTc, supraventricular tachycardia, atrial fibrillation, and other cardiac effects have also been reported in cases of VEN overdose [13,14]. QTc prolongation was not uniformly observed in routine treatment among the elderly [15]. Three of our five patients experienced atrial fibrillation. Although this has not been conclusively linked to VEN, stable sinus rhythm was observed after the reduction of VEN dosage, suggesting that VEN toxicity is a plausible cause.

### 4.1. Pharmacokinetic and Pharmacogenetic Considerations

Assuming partial dose dependency of VEN-associated CTOX, the role of VEN serum concentrations requires discussion. These concentrations are primarily dependent on dose, hepatic cytochrome P450 enzyme activity, and renal function. VEN is eliminated via hepatic metabolism to its major active metabolite, O-Desmethyl-VEN, with subsequent further metabolism involving the cytochrome P450 enzyme subtypes CYP2C19, CYP2D6, and CYP3A4. Urinary excretion accounts for about 45% of the elimination of VEN and its active metabolite, so the VEN dose should be reduced by 50% if creatinine clearance is below 30 mL/min (www.dosing.de/popup_niere.php?monoid=720; accessed on 5 January 2025). Although there are reports of VEN-associated CTOX cardiomyopathy in patients with a CYP2D6 lack of function genotype (poor metabolizer) [16,17,18], no strong correlation has been demonstrated between the CYP2D6 or CYP2C19 genotypes and VEN or O-Desmethyl-VEN plasma levels, nor with the efficacy or safety of VEN therapy. In the one patient for whom we performed CYP2D6 and CYP2C19 genotyping, the results could also not provide an explanation for VEN-associated CTOX. Currently, we see no role for pharmacogenetic testing in clinically useful predictions of VEN-associated CTOX. In contrast, therapeutic drug monitoring (TDM) can be performed in clinical practice, allowing for rational dose adjustments when necessary. This approach controls intra- and interindividual variability in VEN pharmacokinetics and pharmacogenetics. Accordingly, therapeutic drug-monitoring guidelines recommend routine TDM for VEN [19,20]. In our series, none of the five patients with cardiac toxicity due to VEN had drug levels determined by their treating physicians, suggesting an underuse of TDM for VEN dosing. Therefore, we want to emphasize that VEN TDM is likely an effective measure to prevent VEN-associated CTOX.

### 4.2. Differential Diagnosis and Other Etiologies of Reversible Cardiomyopathies

Apart from genetic causes of hypertrophic and dilated cardiomyopathy [21], which include a critical evaluation of variants of unknown significance suggesting a genetic origin, other rare causes must always be considered when evaluating patients with heart failure. These reversible etiologies include pheochromocytoma [22], acromegaly [23], and hyperthyroidism [24], as well as the application of cardiotoxic drugs such as anthracyclines [25]. Even rarer causes of heart failure include hypocalcemic cardiomyopathy [26]. One of the most commonly underestimated causes of reversible cardiomyopathy is cardiomyopathy induced by arrhythmias [27], which may manifest in patients with sinus tachycardia, tachycardic atrial fibrillation, and frequent ventricular premature complexes [28].

Classical iatrogenic heart failure is often induced by right ventricular pacing in pacemaker patients [29].

In addition, for any patient with heart failure of unknown etiology who does not have coronary artery disease or chronic right ventricular pacing, other reversible causes should be considered and, if feasible, addressed. The notable case of patient 2, who presented with significant reversible hypertrophic cardiomyopathy, exemplifies the necessity to consider alternative causes even when patients exhibit a classic hypertrophic cardiomyopathy phenotype. Besides the recently identified VEN cardiomyopathy, other causes of hypertrophy, such as undetected coarctation leading to left ventricular hypertrophy [30], must be ruled out. Additionally, the differential diagnosis of hypertrophic cardiomyopathy encompasses a range of metabolic and multi-organ diseases, including sarcomere disease, RAS-MAPK signaling disease, mitochondrial myopathies, glycogen and lysosomal storage disorders in children, as well as Anderson–Fabry glycogen storage disorder [31], amyloid, sarcoidosis, Danon cardiomyopathy [32], athlete’s heart, and anabolic steroid effects [20].

### 4.3. Comparison of Cardiotoxicity of SNRIs Such as Venlafaxine with SSRI

Selective serotonin reuptake inhibitors (SSRIs), such as citalopram and escitalopram, are widely used antidepressants. Although SSRIs are known to have some degree of cardiotoxicity, this is significantly less pronounced and generally less harmful compared to serotonin–norepinephrine reuptake inhibitors (SNRIs). The cardiotoxic effects of SSRIs primarily include QTc prolongation, which rarely may lead to torsades de pointes, atrial fibrillation, bradycardia, and occasionally hypotension [33].

However, SNRIs such as venlafaxine seem to be more cardiotoxic, particularly because they not only cause QTc prolongation, but also induce hypertension at higher dosages, potentially leading to left ventricular hypertrophy, increased arrhythmias, and even reversible heart failure and hypertrophic cardiomyopathy, as described in recent series—effects which have not been observed with SSRIs. Contrarily, citalopram has even been reported to improve NT-proBNP levels and left ventricular ejection fraction [34].

### 4.4. Recommendations for Clinical Practice

In addition to the routine monitoring of serum levels of venlafaxine and O-desmethyl-VEN, we recommend regular cardiac examinations. These examinations should include blood pressure assessment, an ECG once a year, echocardiography if cardiac symptoms or abnormal ECG findings are present, and measurement of NT-proBNP levels. This approach enables early detection of signs of VEN-associated CTOX and necessary therapeutic adjustments. For patients at high cardiovascular risk—those with known cardiac disease, hypertension, or a proven genetic predisposition to slow metabolization of VEN—we should consider alternative antidepressants that have a lower cardiotoxic potential, such as SSRIs or atypical antidepressant agents.

### 4.5. Limitations

This is an observational series of only five patients, and not all information and investigation results of interest were retrospectively available for all presented patients. As such, this small case series is not suitable for a formal quantitative evaluation of the incidence or potential risk factors of VEN-associated CTOX.

The five patients also had other possible etiologies of left ventricular hypertrophy and/or heart failure, such as arterial hypertension, coronary artery disease, atrial fibrillation, or right ventricular pacing. However, since there was reversibility in all cases, these causes might have had an additional impact aside from VEN.

## 5. Conclusions

The results of this retrospective case analysis suggest that VEN may have significant, albeit reversible, cardiotoxicity, particularly if given long-term and without monitoring of serum levels. The observed cardiovascular changes highlight the necessity for individualized therapy and close surveillance of VEN serum concentrations and signs of cardiotoxicity. Prioritizing patient safety and fostering tailored treatment approaches are essential to minimizing the risk of cardiovascular side effects.

## Figures and Tables

**Table 1:** VEN serum levels and cardiovascular findings before and during VEN treatment in patient #3.

Date	HR (bpm)	Arterial Hypertension (mmHg)	LVEF (%)	GLS (%)	NT-proBNP (ng/L)	VEN Dose Daily (mg)	VEN + O-Desmethyl-VEN Serum Level (µmol/L-Norm: 0.36–1.44 µmol/L)	Arrhythmias
May 2016	65	124/62	61	NA	NA	0	NA	<1% APCs
Aug 2018	80	128/71	50	NA	1547	0	NA	<1% APCs
Oct 2019	101	115/62	50	−13.8	NA	150	NA	Paroxysmal atrial flutter 9% APCs
April 2020	75	121/72	59	−12.1	NA	150	NA	None reported
Oct 2020	87	126/88	27	NA	3492	300	NA	Paroxysmal atrial fibrillation
Oct 2021	73	107/58	66	NA	141	150	0.76	No arrhythmias reported
11 December 2023	96	102/56	42	−10.0	3464	375	6.83	No arrhythmias reported
27 December 2023	68	127/67	62	−12.4	849	150	3.30	No arrhythmias reported
March 2024	82	128/74	56	NA	191	150–300	3.84	No arrhythmias reported

**Legend Table 1.** HR = heart rate; LVEF = left ventricular ejection fraction; GLS = average global left ventricular strain. VEN = venlafaxine; VEN + O-Desmethyl-VEN serum level normal values: 0.36–1.44 µmol/L); NA = not available; APC = atrial premature contractions.

**Table 2:** Cardiovascular parameters before and after VEN dosage reduction in patient #5.

Date	HR (bpm)	LVEF (%)	GLS (%)	NT-proBNP (ng/L)	VEN Dosage (mg)	VEN + O-Desmethyl-VEN Serum Level (µmol/L-Norm: 0.36–1.44 µmol/L)
23 March 2018	70	56	−19.6	641	0	NA
12 September 2019	73	49	−15.4	NA	150	NA
9 January 2020	NA	NA	NA	3345	150	NA
29 October 2021	69	48	−17.8	NA	150	NA
29 November 2021	88	50	−13.6	NA	150	NA
7 December 2023	87	37	−11.2	2096	150	1.98
1 March 2024	77	44	−13.5	1390	75	0.76

Legend Table 2. HR = heart rate; LVEF = left ventricular ejection fraction; GLS = global longitudinal strain; VEN = venlafaxine; NA = not available

**Table 3:** Summary of the duration and dose of VEN therapy, and signs of cardiac toxicity for all 5 patients.

Patient	Age (Years)	Gender	Indication for VEN	Duration of VEN Therapy (Years)	Max. VEN Dose (mg)	Possible Risk Factors for VEN Toxicity	Signs of VEN Cardiac Toxicity
1	52	female	Migraine	1.5	150	Female gender	Hypertension, left ventricular hypertrophy, and ECG changes: all reversible
2	51	female	Anorexia	15	150	Low weight, female gender	Hypertension, left ventricular hypertrophy, and ECG changes
3	77	female	Depression	3	375	High VEN dose, female gender, old age	Heart failure, atrial fibrillation, hypertension
4	82	male	Depression	8	187.5	Chronic renal failure, old age	Heart failure, atrial fibrillation
5	87	male	Depression	4.3	150	Chronic renal failure, old age	Heart failure, atrial fibrillation

Legend Table 3. VEN = venlafaxine.

**Table 4:** Cardiovascular parameters in the 5 patients at the initial presentation and during follow-up.

Patient	Age (Years)	Arterial Hypertension (mmHg)	Abnormal ECG (max. QTc)	Atrial Arrhythmias	LVEF at Diagnosis (%)	LVEF at Last Follow-Up (%)	GLS at Diagnosis (%)	GLS at Last Follow-Up (%)	Blood Pressure at Presentation and Last Follow-Up (mmHg)	QTc-Interval at Diagnosis and Last Follow-Up (ms)
1	52	yes	yes	no	59	59	−15.9	−21	170/80/143/84	470/443
2	51	yes	yes	no	27	62	−10	−12.4	150/85/130/80	451/430
3	77	yes	yes	paroxysmal atrial fibrillation	60	54	−13	−13.8	170/90/150/85	457/416
4	82	yes	yes	atrial fibrillation, sinus rhythm after VEN reduction	20	49	−5.7	−16.7	180/95/160/85	460/430
5	87	yes	Pace-maker	atrial fibrillation, now sinus rhythm	37	44	−11.2	−13.5	160/90/150/85	457/450

Legend Table 4. ECG = electrocardiogram; LVEF = left ventricular ejection fraction; GLS = global longitudinal strain.

## Data Availability

The original contributions presented in this study are included in the article/Supplementary Material. Further inquiries can be directed to the corresponding author(s).

## Supplementary Materials

The following supporting information can be downloaded at: https://www.mdpi.com/article/10.3390/jcm14082792/s1.
